# Effects of certain pre-analytical factors on the performance of plasma phospho-tau217

**DOI:** 10.1186/s13195-024-01391-1

**Published:** 2024-02-08

**Authors:** Divya Bali, Oskar Hansson, Shorena Janelidze

**Affiliations:** 1 Clinical Memory Research Unit, Department of Clinical Sciences, Lund University, Sölvegatan 19, BMC B11, 22184 Lund, Sweden; 2 Memory Clinic, Skåne University Hospital, 20502 Malmö, Sweden

**Keywords:** Blood-based biomarkers, Pre-analytical factors, Alzheimer’s disease, Phosphorylated tau (p-tau)217, Amyloid-β

## Abstract

**Introduction:**

Pre-analytical factors can cause substantial variability in the measurements of cerebrospinal fluid (CSF) and plasma biomarkers of Alzheimer’s disease (AD). However, their effects on the performance of one of the most promising plasma AD biomarkers, phosphorylated tau (p-tau)217, are not known.

**Methods:**

We included 50 amyloid-β positive (Aβ^+^) and 50 Aβ^−^ participants from the Swedish BioFINDER-1 study. Plasma and CSF p-tau217 were measured using an immunoassay developed by Lilly Research Laboratories. We examined the effect of four plasma handling conditions, i.e., (1) thawing at room temperature (RT) with no centrifugation, (2) thawing at RT followed by centrifugation, (3) thawing on ice with no centrifugation, and (4) thawing on ice followed by centrifugation. In addition, we also tested the effects of up to 3 freeze–thaw cycles on the associations of plasma p-tau217 with AD-related pathologies measured with CSF p-tau217 and CSF Aβ42/Aβ40.

**Results:**

In the whole cohort (combining Aβ^+^ and Aβ^−^ participants), we found significant correlations between plasma p-tau217 and both CSF p-tau217 (*R*_range,_ 0.614–0.717, *p* < 0.001) and CSF Aβ42/Aβ40 (Spearman *R*_range_, − 0.515 to − 0.652, *p* < 0.001) for each of the four tested conditions. Correlations between plasma and CSF p-tau217 were also significant for all conditions in the Aβ^+^ group (*R*_range_, 0.506–0.579, *p* < 0.001). However, in this Aβ^+^ subgroup, correlations with CSF Aβ42/Aβ40 were only significant for centrifuged samples (thawed at RT, *R* =  − 0.394, *p* = 0.010; thawed on ice, *R* =  − 0.406; *p* = 0.007). In Aβ^−^ participants, correlations between plasma and CSF p-tau217 were again significant only for centrifuged samples (thawed at RT, *R* = 0.394, *p* = 0.007; thawed on ice, *R* = 0.334; *p* = 0.022), with no correlations seen between plasma p-tau217 and CSF Aβ42/Aβ40 for any of the conditions. While the accuracy of plasma p-tau217 to identify individuals with abnormal CSF Aβ42/Aβ40 or CSF p-tau217 status was high, the AUCs for samples thawed at RT and analyzed without centrifugation were numerically lower than the AUCs of other conditions (CSF Aβ42/Aβ40 = 0.845 vs 0.872–0.884; CSF p-tau217 = 0.866 vs 0.908–0.924, *p*_diff_ > 0.11). P-tau217 concentration was consistently higher in non-centrifuged samples than in centrifuged samples (*p* ≤ 0.021). There were no differences between samples freeze-thawed once, twice, or three times.

**Conclusion:**

Centrifugation improved the performance of plasma p-tau217, but thawing temperatures and up to three freeze–thaw cycles did not have a significant impact. These results may inform the future development of standardized sample-handling protocols for AD biomarkers.

**Supplementary Information:**

The online version contains supplementary material available at 10.1186/s13195-024-01391-1.

## Introduction

Alzheimer’s disease (AD), the most prevalent cause of dementia, is an escalating neurodegenerative disease. According to the World Health Organization (WHO), globally more than 55 million people are living with dementia and approximately 10 million people develop the disease every year [1]. The estimated cost for treating AD and other dementias is $345 billion in 2023, and with aging population, this cost is expected to reach around $1 trillion by 2050 [2]. This poses a huge economic challenge on healthcare. AD-related amyloid-β (Aβ) and tau pathologies are detectable and monitored in cerebrospinal fluid (CSF) and using positron emission tomography (PET) with high accuracy; however, their broad implementation is impractical in routine clinical care practices [3, 4]. Therefore, the need of the hour is blood-based biomarkers—an easily accessible, minimally invasive, and cost-effective method; which can be widely employed in both clinical trials and clinical settings [5].

Plasma phosphorylated tau (p-tau)217 emerged as one of the most promising blood-based biomarkers for AD [5]. Plasma p-tau217 levels are elevated in the preclinical stage of AD and continue to rise in the mild cognitive impairment (MCI) and dementia stages [6–8]. Interestingly, some studies have reported that plasma p-tau217 exhibited better accuracy for detecting abnormal PET and CSF status, distinguishing AD from other neurological disorders, and when predicting future progression to AD dementia in MCI patients in comparison to plasma p-tau181 [8–11]. A recent work demonstrated that plasma levels of p-tau217 but not other candidate plasma AD biomarkers (e.g., p-tau181 and p-tau231) clearly increase over time in people with abnormal brain Aβ deposition correlating with brain atrophy and cognitive decline [12].

Previous studies have shown that preanalytical variables can greatly influence the concentration and performance of CSF AD biomarkers [13]. As a result, a standardized protocol for handling CSF samples prior to AD biomarker measurements has been established [13–15] aiming to reduce variability in results and thereby allow comparisons of biomarker data across various laboratories as well as the establishment of universal cutoff values. Several studies have examined the effects of various pre-analytical factors on the plasma levels of p-tau181. Plasma p-tau181 levels quantified with single molecule assays (Simoa) were found to decline after three freeze–thaw cycles [16]. At the same time, other studies reported that levels of plasma p-tau181 assessed with Simoa [17] or Elecsys Roche immunoassays [18] were stable up to four repeated freeze–thaw cycles and were not influenced by delayed centrifugation, centrifugation temperature, differences in aliquot volumes, delayed post-centrifugation even after 4-h and 24-h storage at room temperature or in the fridge (2–8 °C) [17, 19], and 2-week intermediate storage at − 20 °C or at 2–8 °C. Another recent study demonstrated that plasma p-tau181 concentration quantified using the LUMIPULSE-GS600 II automated platform decreased when stored at 4 °C for either 1–2 days or 8–9 days in comparison to storing at − 20 °C [20]. To date, only one study assessed the effects of pre-analytical factors on plasma p-tau217 showing that its levels were unaltered by up to four freeze–thaw cycles, choice of anticoagulant, plastic tube types, and transfer of plasma sample from one tube to another [18]. However, in this study, plasma p-tau217 was measured using Elecsys Roche plasma prototype immunoassay that uses different antibodies and platform than the better-performing plasma p-tau217 assay on Meso Scale Discovery (MSD) platform developed by Lilly Research laboratories [21, 22]. Of note, all the previous studies only tested the impact of pre-analytical factors on plasma concentrations of p-tau biomarkers and in a relatively small sample. The aim of the present study was to examine the effects of the pre-analytical factors such as thawing conditions, centrifugation, and freeze–thaw cycles on the levels of plasma p-tau217 and most importantly on associations of plasma p-tau217 with AD-related brain Aβ and tau pathologies. To this end, we measured plasma p-tau217 using the high-performing Lilly p-tau217 immunoassays in 50 amyloid-β positive (Aβ^+^) and 50 Aβ^−^ individuals.

## Materials and methods

### Study participants

This study included 100 participants from the Swedish BioFINDER-1 study of whom 50 were CSF Aβ42/Aβ40 positive (Aβ^+^) and 50 were CSF Aβ42/Aβ40 negative (Aβ^−^). All participants were recruited at Skåne University Hospital and the Hospital of Ängellholm. Detailed information about the recruitment process and inclusion/exclusion criteria has been previously described [23, 24]. The demographics of study participants are shown in Table 1.

### Blood collection and handling

**Fig. 1:**
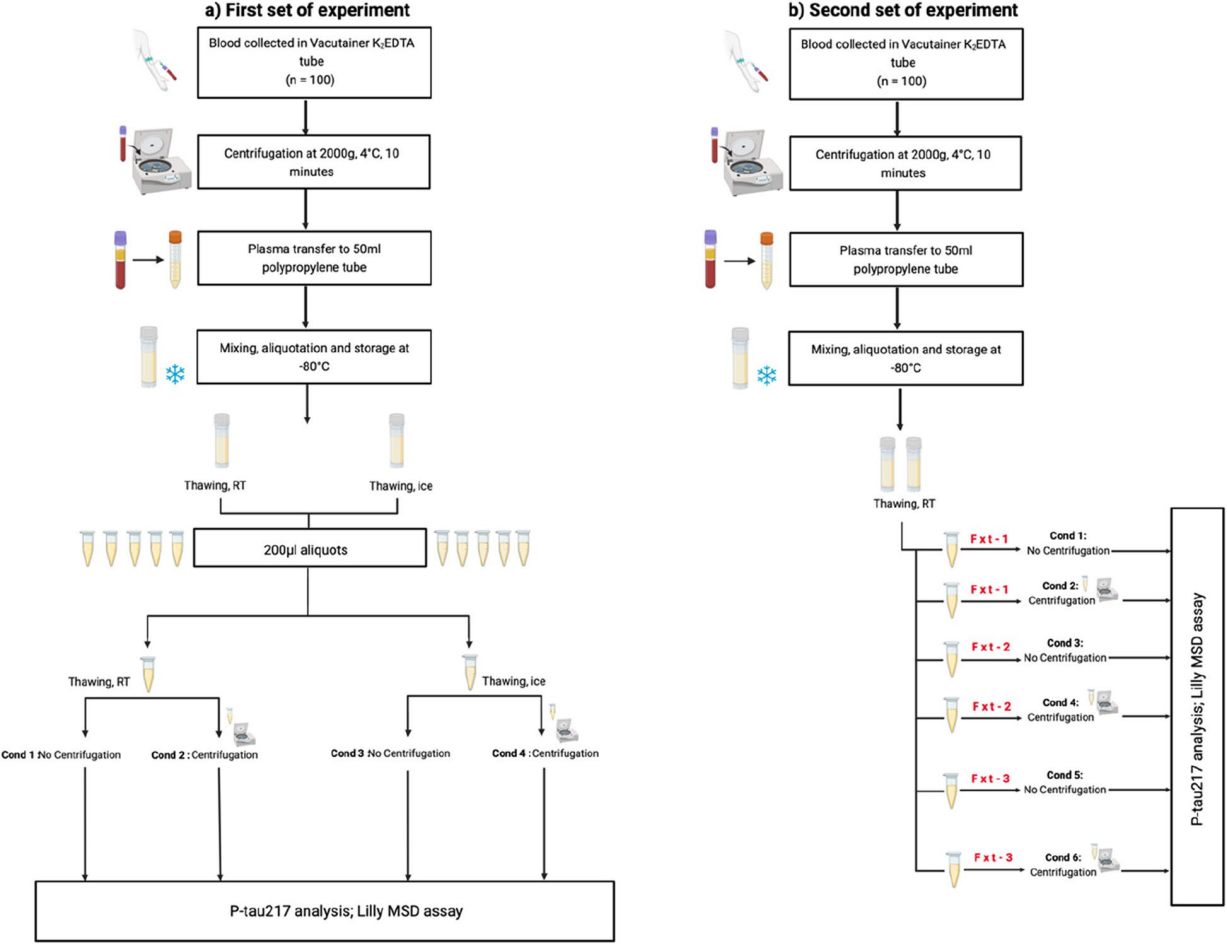
Schematic representation of pre-analytical sample handling conditions. **a** The blood was collected in 6 EDTA tubes from each of the 100 study participants and centrifuged at 2000* g* at 4 °C for 10 min. After centrifugation, plasma was transferred from all six EDTA tubes into one 50-ml tube, mixed, and 1 ml was aliquoted into 1.5-ml tubes and stored at − 80°C within 30 min after collection. For each participant, two tubes with 1 ml of frozen plasma were used. One frozen plasma tube was thawed on ice while the other tube was thawed at RT. Following thawing, 200-μl plasma aliquots were prepared. For samples thawed at RT, one 200-μl plasma aliquot was centrifuged at RT (10 min, 2000* g*) whereas another 200-μl plasma aliquot was not centrifuged and stored at RT until the p-tau217 analysis. The plasma aliquots prepared from the tube thawed on ice were treated in the same way except that centrifugation and sample storage prior to the p-tau217 analysis were performed at + 4 °C and on ice respectively. **b** To assess the effect of freeze–thaw cycles, a new 1-ml plasma tube was used for each participant. The frozen plasma tube was thawed at RT and four 70-μl plasma aliquots were prepared. Two of the four plasma aliquots did not undergo any further freeze–thaw cycles and were thus fxt-1; the other two 70-μl plasma aliquots underwent one additional freeze–thaw cycle (fxt-2) within 30 min prior to analysis. Additionally, two plasma aliquots that were fxt-3 times (prepared from the previous set of experiments) were also included. One aliquot of each of the three freeze–thaw conditions (fxt-1, 2, or 3) was centrifuged at RT whereas another aliquot was not centrifuged and stored at RT until the p-tau217 analysis. Abbreviations: Cond, Condition; EDTA, ethylenediaminetetraacetic acid; fxt, freeze–thaw cycle; RT, room temperature. Created with BioRender.com

Blood samples from non-fasting participants were collected in the morning. For plasma extraction, blood from each participant was collected in six Ethylenediaminetetraacetic acid (EDTA) tubes (Vacutainer K_2_EDTA tube; BD Diagnostics) and centrifuged (2000* g*, 4 °C) for 10 min (Fig. 1). After centrifugation, plasma was transferred from all six EDTA tubes into one 50-ml tube, mixed, and 1 ml was aliquoted into 1.5-ml tubes and stored at − 80 °C within 30 min after collection.

### Pre-analytical sample handling procedures

#### Thawing temperatures and centrifugation

In the first set of experiments, we studied the effects of thawing temperatures (room temperature (RT) and ice) and centrifugation (Fig. 1a). For each participant, two tubes with 1 ml of plasma were used. One frozen plasma tube was thawed at RT, while the other tube was thawed on ice. Following thawing, 200 μl plasma aliquots were prepared. For samples thawed at RT, one 200-μl plasma aliquot was centrifuged at RT (10 min, 2000* g*) whereas another 200 μl plasma aliquot was not centrifuged and stored at RT until the p-tau217 analysis. The plasma aliquots prepared from the tube thawed on ice were treated in the same way except that centrifugation and sample storage prior to the p-tau217 analysis were performed at + 4 °C and on ice respectively. Thus, four different plasma handling conditions were tested, i.e., (1) thawing at RT, no centrifugation; (2) thawing at RT, centrifugation; (3) thawing on ice, no centrifugation; and (4) thawing on ice, centrifugation.

#### Freeze–thaw cycles

In the second set of experiments, we assessed the effects of freeze–thaw cycles (Fig. 1b). For each participant, one new tube with 1 ml of plasma was used. The frozen plasma tubes were thawed at RT and four 70-μl aliquots were prepared. Two of the four plasma aliquots did not undergo any further freeze–thaw cycles and were thus freeze-thawed once; the other two 70-μl plasma aliquots underwent one additional freeze–thaw cycle (freeze-thawed twice) within 30 min before the start of the p-tau217 assays. Our unpublished data indicated that plasma p-tau217 concentration changes very little (by 5.7% on average) after storage for 30 min at RT. For each participant, we also included two plasma aliquots that were freeze-thawed three times (these aliquots were prepared in the previous set of experiments). One aliquot of each of the three freeze–thaw conditions (freeze-thawed once, twice, or thrice) was centrifuged at RT (10 min, 2000* g*) whereas another aliquot of each of the three freeze–thaw conditions (freeze-thawed once, twice, or thrice) was not centrifuged and stored at RT until the p-tau217 analysis.

### CSF collection, processing, and handling

CSF samples were collected at the same time as plasma samples. CSF was obtained via lumbar puncture and stored at − 80 °C in polypropylene tubes following the Alzheimer’s Association flow chart for lumbar puncture and CSF sample processing [25]. CSF from each participant was collected in two 10-ml tubes and centrifuged for 10 min (2000* g*, 4 °C). Following centrifugation, 1 ml was aliquoted into 1.5-ml LoBind tubes and stored at − 80 °C within 30 min after collection. All CSF samples went through one freeze–thaw cycle before the analysis.

### Plasma and CSF analysis

Plasma p-tau217 and CSF p-tau217 concentrations were measured using an immunoassay developed by Lilly Research Laboratories on the MSD platform at the Memory Research Unit, Lund University, as previously described [8, 26]. Briefly, the small-spot streptavidin-coated MSD plate was incubated with 25 μl of 0.5 μg/ml anti-p-tau217 capture antibody (biotinylated-IBA493) per well for 1 h at RT. Then, 50 μl of calibrators and diluted samples were added to each plate and incubated for 2 h at RT on a shaker at 650 rpm. Finally, the plate was incubated with 25μl of 0.02 μg/ml anti-tau detection antibody (SULFO-TAG-4G10-E2) per well for 1 h at RT. All incubations were performed on a shaker at 650 rpm. In the final step, the plate was read on the MSD SQ120 plate reader. The immunoassay was calibrated using synthetic p-tau217 peptide. The plasma and CSF samples were diluted by a factor of 2 and 4, respectively. CSF Aβ42/Aβ40 levels were measured using Elecsys® electrochemiluminescence immunoassays on a fully automated cobas e 601 instrument (Roche Diagnostics International Ltd.). For CSF Aβ42/Aβ40, we used a previously established threshold of 0.66 [21], and for CSF p-tau217, the cut-off (11.9) was calculated using the mean plus 2 standard deviations of a large group of cognitively unimpaired Aβ^−^ individuals (*n* = 403) from the BioFINDER-1 study. The average intra-plate (Figure S1) and inter-plate coefficients of variability were 6.98% (8.68% Aβ^−^, 5.28% Aβ^+^) and 7.91%, respectively, and the average limit of the detection of the assay was 0.16 pg/ml.

### Statistical analysis

SPSS version 28 (IBM) and R version (2022.12.0 + 353) were used to perform all the statistical analysis and the data was visualized using GraphPad Prism version 8. Group differences in demographic and clinical data and plasma p-tau217 levels were examined using Mann–Whitney *U* and chi-square (sex and APOE positivity) tests. The Spearman test was used to determine correlations between CSF biomarkers and plasma p-tau217. The 95% confidence intervals (CIs) estimated from 2000 bootstrap iterations were used to test differences in the correlation coefficients. The accuracy of plasma p-tau217 to distinguish abnormal from normal CSF Aβ42/Aβ40 or p-tau217 status was assessed using receiver operating characteristic curve (ROC) analysis. The Youden index was used to determine the sensitivity and specificity with a 95% confidence interval at the optimal threshold value. The DeLong test was used to determine whether the area under the curve (AUC) of two ROC curves was statistically different. The effects of different sample handling conditions on plasma p-tau217 concentration were examined with a 2-way repeated measures of variance (ANOVA) including sample handling conditions, Aβ status, and their interaction as independent variables. *P*-values were corrected for multiple comparisons using Benjamini Hochberg’s false discovery rate; *p* < 0.05 was considered statistically significant.

## Results

### Participants

We included 50 Aβ^+^ (35 cognitively unimpaired (CU), 6 mild cognitive impairment (MCI), 8 Alzheimer disease dementia (ADD), and 1 vascular dementia (VaD)) and 50 Aβ^−^ participants (48 CU, 1 VaD, and 1 normal pressure hydrocephalus (NPH)) (Table 1). There was no difference in sex (*p* = 0.53) and education (*p* = 0.58), whereas MMSE score was lower (*p* = 0.005); *APOE* $\varepsilon 4$ rate (*p* = 0.001) and CSF p-tau217 levels (*p* < 0.001) were higher, whereas (by definition) CSF Aβ42/Aβ40 levels (*p* < 0.001) were lower in the Aβ^+^ group.

**Table 1:** Participant characteristics

	Total (*n* = 100)	Aβ^+^ (*n* = 50)	Aβ^−^ (*n* = 50)	*P*-value Aβ^−^ vs Aβ^+^
Age (year)	77.5 (80–40)	77.5 (80–74.75)	77.5 (81–73.75)	0.967
Diagnosis CU/MCI/ADD/VaD/ NPH, n	83/6/8/2/1	35/6/8/1/0	48/0/0/1/1	0.002
Sex (female/male), *n*	63/37	33/17	30/20	0.534
Education, years	11 (13.75–9)	11 (13.25–9)	12 (14–9.75)	0.577
MMSE score	28 (29–27)	28 (29–26)	29 (29.25–28)	0.005
*APOE* $\varepsilon 4$ status pos./neg. (%pos.)	38/61 (38.4%)	29/21 (58%)	9/40 (18.4%)	0.001
CSF p-tau217, pg/ml	9.44 (21.89–5.27)	21.68 (34.84–13.93)	5.73 (7.91–4.42)	< 0.001

Data are shown as median (interquartile range). Group differences were analyzed using the Mann–Whitney test. For sex and *APOE* $\varepsilon 4$ status, the chi-square test was used. *Abbreviations: Aβ*^+^ Amyloid-β positive, *Aβ*^*−*^ Amyloid-β negative, *ADD* Alzheimer disease dementia, *APOE* Apolipoprotein E, *CSF* Cerebrospinal fluid, *CU* Cognitively unimpaired, *MCI* Mild cognitive impairment, *MMSE* Mini-Mental State Examination, *NPH* Normal pressure hydrocephalus, *VaD* Vascular dementia

### Effects of pre-analytical conditions on p-tau217

#### Association of plasma p-tau217 with amyloid pathology

**Fig. 2:**
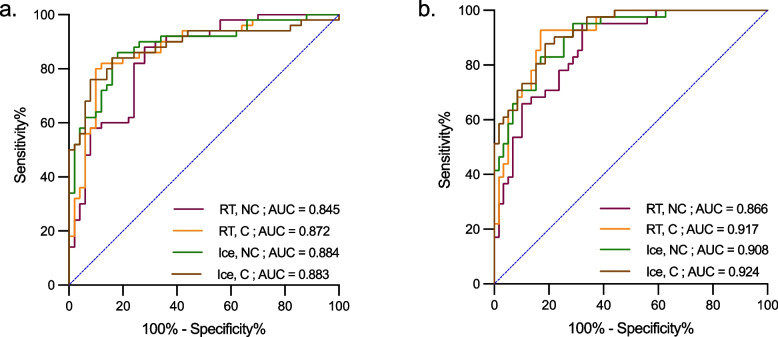
The accuracy of plasma p-tau217 to identify individuals with abnormal CSF Aβ42/Aβ40 or p-tau217 status. ROC curve analyses for identifying abnormal **a** CSF Aβ42/Aβ40 status and **b** CSF p-tau 217 status (see Tables 3 and 5 for sensitivity and specificity measures). Abbreviations: AUC, area under the curve; C, centrifugation; NC, non-centrifugation; ROC, receiver operating characteristic; RT, room temperature

We first examined how pre-analytical sample handling conditions such as thawing at RT or on ice and centrifugation or no centrifugation (Fig. 1a) affected the association between plasma p-tau217 and CSF Aβ42/Aβ40 (Table 2). In the whole cohort, there were significant negative correlations between plasma p-tau217 and CSF Aβ42/Aβ40 for all four sample handling conditions (*R*_range_, − 0.515 to − 0.652; *p* < 0.001). We did not find significant differences between the correlation coefficients (*p* > 0.08). In the Aβ^+^ group, the correlations were significant in centrifuged samples (thawed at RT: *R*, − 0.394; *p* = 0.010; thawed on ice: *R*, − 0.406; *p* = 0.007) but not in non-centrifuged samples. As expected, no correlations were seen in the Aβ^−^ group. In ROC analysis, when testing the accuracy of plasma p-tau217 to distinguish abnormal from normal CSF Aβ status (Fig. 2, Table 3, Table S1), we found high AUCs for all sample handling conditions (AUC range, 0.845–0.884). While there were no significant differences between the AUCs (*p* > 0.58), the non-centrifuged samples that were thawed at RT showed numerically lower performance (AUC, 0.845) compared with other conditions (AUC range, 0.872–0.884).

**Table 2:** Spearman correlations between plasma p-tau217 and CSF Aβ42/Aβ40

Plasma p-tau217	CSF Aβ42/Aβ40 *R* (*p*-value, adjusted/unadjusted)
All (*n* = 100)	
Cond 1: thaw at RT, NC	** − 0.515 (< 0.001/ < 0.001)**
Cond 2: thaw at RT, C	** − 0.636 (< 0.001/ < 0.001)**
Cond 3: thaw on ice, NC	** − 0.607 (< 0.001/ < 0.001)**
Cond 4: thaw on ice, C	** − 0.652 (< 0.001/ < 0.001)**
Aβ^+^ (*n* = 50)	
Cond 1: thaw at RT, NC	− 0.215 (0.172/0.133)
Cond 2: thaw at RT, C	** − 0.394 (0.010/0.005)**
Cond 3: thaw on ice, NC	− 0.284 0.079/0.046)
Cond 4: thaw on ice, C	** − 0.406 (0.007/0.003)**
Aβ^−^ (*n* = 50)	
Cond 1: thaw at RT, NC	0.230 (0.162/0.108)
Cond 2: thaw at RT, C	0.073 (0.615/0.615)
Cond 3: thaw on ice, NC	0.210 (0.172/0.143)
Cond 4: thaw on ice, C	0.105 (0.511/0.468)

Data are shown as Spearman correlation coefficients (*p*-values, adjusted/unadjusted) with significant results highlighted in bold. *Abbreviations: Aβ*^+^ Amyloid-β positive, *Aβ*^*−*^ Amyloid-β negative, *C* Centrifugation, *Cond* Condition, *CSF* Cerebrospinal fluid, *NC* Non-centrifugation, *RT* Room temperature

**Table 3:** ROC analysis of plasma p-tau 217 for identifying abnormal CSF Aβ42/Aβ40 status

Plasma p-tau217	AUC (95% CI)	Sensitivity %	Specificity %	Cut-off	Youden’s Index
Cond 1: thaw at RT, NC	0.845 (0.769, 0.921)	88	72	0.282	.600
Cond 2: thaw at RT, C	0.872 (0.799, 0.945)	80	90	0.247	.700
Cond 3: thaw on ice, NC	0.884 (0.818, 0.951)	86	82	0.252	.680
Cond 4: thaw on ice, C	0.883 (0.813, 0.953)	76	92	0.256	.680

*Abbreviations: Aβ*^+^ Amyloid-β positive, *Aβ*^*−*^ Amyloid-β negative, *AUC* Area under the curve, *CI* Confidence interval, *CSF* Cerebrospinal fluid, *C* Centrifugation, *Cond* Condition, *NC* Non-centrifugation, *ROC* Receiver operating characteristic, *RT* Room temperature

#### Association of p-tau217 in plasma vs its level in CSF

We next investigated the effects of different pre-analytical sample handling conditions on the association between plasma p-tau217 and CSF p-tau217. We found that plasma p-tau217 and CSF p-tau217 were correlated in the whole cohort (*R* range, 0.614–0.717; *p* < 0.001) and in the Aβ^+^ group (*R* range, 0.506–0.579; *p* < 0.001) for all sample handling conditions (Table 4). There were no significant differences between the correlation coefficients (*p* > 0.30). Interestingly, in the Aβ^−^ group, significant correlations were only observed for centrifuged samples (thawed at RT: *R*, 0.394; *p* = 0.007; thawed on ice: Rs, 0.334; *p* = 0.022). In ROC analysis, when evaluating the accuracy of plasma p-tau217 to identify individuals abnormal CSF p-tau217 status, we found high AUCs (range, 0.866–0.924) for all the sample handling conditions (Fig. 2, Table 5, Table S2). Even though there were no significant differences in AUCs between different conditions AUCs (*p* > 0.11), non-centrifuged samples that were thawed at RT again showed numerically lower performance (AUC, 0.866) compared with other conditions (AUC range, 0.908–0.924).

**Table 4:** Spearman correlations between plasma p-tau217 and CSF p-tau217

Plasma p-tau217	CSF p-Tau217 *R* (*p*-value, adjusted/unadjusted)
All (*n* = 100)	
Cond 1: thaw at RT, NC	**0.614 (< 0.001/ < 0.001)**
Cond 2: thaw at RT, C	**0.713 (< 0.001/ < 0.001)**
Cond 3: thaw on ice, NC	**0.666 (< 0.001/ < 0.001)**
Cond 4: thaw on ice, C	**0.717 (< 0.001/ < 0.001)**
Aβ^+^ (*n* = 50)	
Cond 1: thaw at RT, NC	**0.506 (< 0.001/ < 0.001)**
Cond 2: thaw at RT, C	**0.579 (< 0.001/ < 0.001)**
Cond 3: thaw on ice, NC	**0.511 (< 0.001/ < 0.001)**
Cond 4: thaw on ice, C	**0.550 (< 0.001/ < 0.001)**
Aβ^−^ (*n* = 50)	
Cond 1: thaw at RT, NC	0.190 (0.202/0.186)
Cond 2: thaw at RT, C	**0.394 (0.007/0.005)**
Cond 3: thaw on ice, NC	0.184 (0.202/0.202)
Cond 4: thaw on ice, C	**0.334 (0.022/0.018)**

Data are shown as Spearman correlation coefficients (*p*-values, adjusted/unadjusted) with significant results highlighted in bold. *Abbreviations: Aβ*^+^ Amyloid-β positive, *Aβ*^*−*^ Amyloid-β negative, *C* Centrifugation, *Cond* Condition, *CSF* Cerebrospinal fluid, *NC* Non-centrifugation, *RT* Room temperature

**Table 5:** ROC analysis of plasma p-tau217 for identifying abnormal CSF p-tau217 status

Plasma p-tau217	AUC (95% CI)	Sensitivity %	Specificity %	Cut-off	Youden’s Index
Cond 1: thaw at RT, NC	0.866 (0.797, 0.935)	95.1	67.8	0.282	0.629
Cond 2: thaw at RT, C	0.917 (0.864, 0.970)	92.7	83.1	0.240	0.757
Cond 3: thaw on ice, NC	0.908 (0.853, 0.964)	95.1	71.2	0.232	0.663
Cond 4: thaw on ice, C	0.924 (0.875, 0.972)	87.8	81.4	0.242	0.692

*Abbreviations: AUC* Area under the curve, *CSF* Cerebrospinal fluid, *C* Centrifugation, *Cond* Condition, *NC* Non-centrifugation, *ROC* Receiver operating characteristic, *RT* Room temperature

### Plasma p-tau217 levels in Aβ^+^ and Aβ^−^ groups

**Fig. 3:**
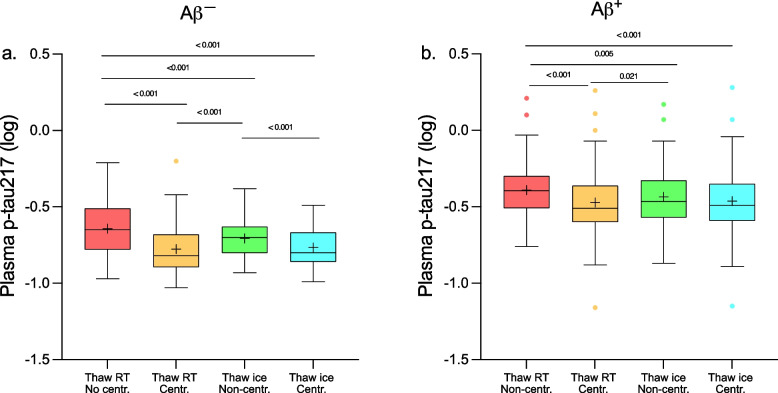
Plasma p-tau217 concentrations in different pre-analytical sample handling conditions. P-tau217 concentrations in EDTA plasma samples that were thawed at RT and analyzed without centrifugation (Cond 1), thawed at RT and centrifuged before the analysis (Cond 2), thawed on ice and analyzed without centrifugation (Cond 3), thawed on ice and centrifuged before analysis (Cond 4). Plasma samples were collected from (**a**) 50 Aβ^−^ and (**b**) 50 Aβ^+^ participants. *P*-values are from the two-way ANOVA repeated measures with FDR correction for multiple comparisons, boxes show interquartile range, the horizontal lines are the medians, and the whiskers are plotted using the Tukey method. Abbreviations: Aβ^+^, amyloid-β positive; Aβ^−^, amyloid-β negative; EDTA, ethylenediaminetetraacetic acid; RT, room temperature; NC, non-centrifugation; C, centrifugation

We also tested the effects of thawing temperatures and centrifugations on plasma p-tau217 concentrations and whether these effects differed between participants with abnormal and normal CSF Aβ42/Aβ40 (Fig. 3, Table 6, and Table S3). There was a significant interaction between Aβ status and sample handling conditions on plasma p-tau217 levels (*F* (3, 294) = 3.176, *p* = 0.025). Specifically in samples thawed on ice, centrifugation led to reduced plasma p-tau217 levels in Aβ^−^ (mean difference (Δ) = 0.06; *p* < 0.001) but not in Aβ^+^ participants. At the same time, in all other comparisons, p-tau217 levels were higher in plasma samples that were not centrifuged compared with those that were centrifuged in both Aβ^+^ and Aβ^−^ groups (Δ_range_ = 0.04 to 0.14; *p* ≤ 0.021).

**Table 6:** Plasma p-tau217 concentrations across different pre-analytical handling conditions

CSF Aβ42/Aβ40	Total (*n* = 100)	Aβ^+^ (*n* = 50)	Aβ^−^ (*n* = 50)	*P*-value Aβ^−^ vs Aβ^+^
Plasma p-tau217, pg/ml				
Cond 1: thaw at RT, NC	0.31 (0.42–0.20)	0.40 (0.51–0.31)	0.22 (0.31–0.17)	< 0.001
Cond 2: thaw at RT, C	0.23 (0.32–0.14)	0.31 (0.43–0.25)	0.15 (0.21–0.13)	< 0.001
Cond 3: thaw on ice, NC	0.26 (0.35–0.19)	0.34 (0.47–0.27)	0.20 (0.23–0.16)	< 0.001
Cond 4: thaw on ice, C	0.23 (0.33–0.16)	0.32 (0.44–0.26)	0.16 (0.21–0.14)	< 0.001
Plasma p-tau217, pg/ml				
Cond 1: thaw at RT, NC, fxt-1	0.30 (0.42–0.22)	0.37 (0.48–0.30)	0.22 (0.28–0.16)	< 0.001
Cond 2: thaw at RT, C, fxt-1	0.19 (0.28–0.15)	0.27 (0.34–0.21)	0.16 (0.18–0.12)	< 0.001
Cond 3: thaw at RT, NC, fxt-2	0.31 (0.39–0.22)	0.36 (0.47–0.31)	0.23 (0.30–0.17)	< 0.001
Cond 4: thaw at RT, C, fxt-2	0.19 (0.29–0.15)	0.27 (0.35–0.20)	0.16 (0.19–0.13)	< 0.001
Cond 5: thaw at RT, NC, fxt-3	0.31 (0.42–0.23)	0.40 (0.47–0.32)	0.24 (0.30–0.19)	< 0.001
Cond 6: thaw at RT, C, fxt-3	0.21 (0.27–0.16)	0.27 (0.37–0.22)	0.17 (0.20–0.14)	< 0.001

Data are shown as median (Interquartile range). Group differences were analyzed using the Mann–Whitney test. *Abbreviations: Aβ*^+^Amyloid-β positive, *Aβ*^*−*^ Amyloid-β negative, *C* Centrifugation, *Cond* Condition, *fxt* Freeze–thaw cycle, *NC* Non-centrifugation, *RT* Room temperature

### Effects of freeze–thaw cycles on plasma p-tau217 concentration

Given that no differences in p-tau217 performance were seen between samples thawed at RT and on ice and that thawing at RT is easier to implement in clinical settings, we assessed the impact of freeze–thaw cycles on plasma p-tau217 levels in samples thawed at RT (centrifuged or non-centrifuged). There was no significant effect of interaction between Aβ status and sample handling conditions (6 different groups shown in Fig. 1b and Figure S2) on plasma p-tau217 levels. Similar to the results above, p-tau217 concentrations were decreased in centrifuged samples compared to non-centrifuged samples (Δ_range_ = 0.13 to 0.17; *p* < 0.001) (Figure S2, Table 6, and Table S4). However, there were no differences in plasma p-tau217 levels between samples that were freeze-thawed once, twice, or three (Figure S2, Table 6, and Table S4). Furthermore, no differences were found in correlation and ROC analyses for samples that were freeze-thawed once compared to those that were freeze-thawed twice or three times (Figure S3 and Table S5-S8). The overall results of the correlation and ROC analyses and the differences between centrifuged and non-centrifuged samples were similar to those seen for samples that were thawed at RT presented in the previous results section.

## Discussion

The Alzheimer’s Association’s international working group has recently recommended the cautious use of blood-based biomarkers followed by confirmatory PET or CSF assessments for both screening of participants in clinical trials and diagnostic workup of patients with cognitive complaints in specialized memory clinics [27]. P-tau217 is considered one of the most accurate blood-based biomarkers of AD, and in the present study, we investigated the effects of pre-analytical factors on its performance aiming to establish robust and easily manageable sample handling conditions that could facilitate future implementation of this biomarker in clinical care and drug trials. We found better correlations of plasma p-tau217 with CSF p-tau217 and Aβ42/Aβ40 for centrifuged samples compared to non-centrifuged samples. Correlations between plasma and CSF p-tau217 were significant for all tested conditions in the whole cohort and in the Aβ^+^ group. However, in Aβ^−^ participants, correlations were only significant in centrifuged but not non-centrifuged samples. Furthermore, while plasma p-tau217 correlated with CSF Aβ42/Aβ40 in the whole cohort for all tested conditions, significant correlations were seen in Aβ^+^ only for centrifuged samples. In ROC analysis, plasma p-tau217 accurately detected abnormal CSF Aβ42/Aβ40 and p-tau217 status. The AUCs were high in all the sample handling conditions with non-centrifuged samples exhibiting numerically lower AUCs. Finally, we also show that thawing conditions (RT or on ice) as well as up to three freeze–thaw cycles did not affect the performance of plasma p-tau217.

Centrifugation of plasma samples prior to analysis is recommended in several but not all immunoassays for AD biomarkers [18]. It is currently unclear whether centrifugation is necessary for p-tau assays on the MSD platform. Here we demonstrate that centrifugation enhances the performance of p-tau217 even though p-tau217 concentration was higher in non-centrifuged samples than in centrifuged samples. These findings suggest that centrifugation likely reduces non-specific signal in the assay, as the associations of plasma p-tau217 with CSF p-tau217 and Aβ42/Aβ40 were stronger in centrifuged samples.

Previous research has reported that thawing frozen plasma on ice (1–6 °C) results in the formation of cryoprecipitates [28], which we speculated may interfere with the determination of plasma p-tau217 levels. However, in the present study, we did not find any differences in the performance of p-tau217 measured in samples thawed at RT or on ice. Thus, our results indicate that thawing samples at RT which is faster and more practical in clinical settings than thawing on ice should be recommended for the plasma p-tau217 assay.

CSF t-tau and p-tau181 concentrations have been shown to remain stable for up to 4 freeze–thaw cycles [29, 30]. At the same time, the effects of freeze–thaw cycles on plasma p-tau appears to be assay-specific. Plasma p-tau181 levels quantified with an in-house SIMOA assay developed at the University of Gothenburg [16] have been reported to decrease after 3 freeze–thaw cycles. Conversely, two other studies have demonstrated that plasma levels of p-tau181 and p-tau217 measured using Elecsys prototype immunoassays [18] and p-tau181 measured using a SIMOA Prototype assay [17] remained stable up to 4 freeze–thaw cycles. In line with the prior research, we observed that the concentration of p-tau217 and its performance were unchanged for up to three freeze–thaw cycles.

To the best of our knowledge, previous studies have only investigated the influence of pre-analytical factors on plasma concentration of AD biomarkers (but not on their performance) and in a relatively small sample. One strength of the present study is a large sample size comprising both Aβ^+^ and Aβ^−^ participants. This enabled us to investigate how pre-analytical factors impact the performance of p-tau217 and uncover important effects of centrifugation that were only seen in the Aβ^+^ and Aβ^−^ groups but not in the whole cohort. Our findings highlight the need to include large cohorts of individuals with and without AD pathology and to investigate the impact of pre-analytical variables on biomarkers performance rather than only on their concentrations in future studies on sample handling procedures. There are some limitations to our study. We only tested EDTA and no other tube types such as lithium heparin (LiHep), citrate, or serum. EDTA tubes have been frequently used for blood sampling and hence our results are highly relevant especially for the analysis of already collected and stored EDTA plasma. We did not examine how storage conditions might affect the levels of plasma p-tau217 which is of importance for the measurements of p-tau217 in longitudinal samples. Finally, all plasma samples underwent at least one freeze–thaw cycles. Even though there was no difference between samples freeze-thawed once, twice, or thrice, future studies should compare p-tau217 measured in freshly collected vs freeze-thawed samples. Furthermore, plasma levels of p-tau217 were quantified using MSD platforms and the applicability of our findings to other analytical methods needs to be further investigated.

In conclusion, our findings suggest that optimal sample handling conditions for p-tau217 quantification are the thawing of frozen plasma at RT and centrifugation immediately prior to analysis. These results would be important for the development of a standardized protocol for pre-analytical sample handling and future implementation of p-tau217 in clinical practice and drug trials.

### Supplementary Information

**Additional file 1:** **Fig S1.** Intra-assay precision of plasma p-tau217 Lilly MSD assay. **Fig S2. **Plasma p-tau217 in different pre-analytical sample handling conditions. **Fig S3. **The accuracy of plasma p-tau217 to idetntify individuals with abnormal CSF Aβ42/Aβ40 or p-tau217 status. **Table S1. **Applying Youden-based cutoff of a reference condition (condition 4)^a^  to determine accuracies, sensitivities and specificities of other conditions when identifying abnormal CSF Aβ42/Aβ40 status. **Table S2.** Applying Youden-based cutoff of a reference condition (condition 4)^a^  to determine accuracies, sensitivities and specificities of other conditions when identifying abnormal CSF p-tau217 status. **Table S3. **Mean Differences in plasma p-tau217 levels between reference condition (condition 4)^a^ and other conditions, first set of experiments. **Table S4. **Mean Differences in plasma p-tau217 levels between reference condition (Condition 2)^a^ and other conditions, second set of experiments. **Table S5. **Spearman correlations between plasma p-tau217 and CSF Aβ42/Aβ40. **Table S6. **ROC analysis of plasma p-tau 217 for identifying abnormal CSF Aβ42/Aβ40 status. **Table S7. **Spearman correlations between plasma p-tau217 and CSF p-tau217. **Table S8. **ROC analysis of plasma p-tau 217 for identifying abnormal CSF p-tau217 status.

## Data Availability

Pseudonymized data will be shared by request from a qualified academic investigator for the sole purpose of replicating procedures and results presented in the article and as long as data transfer is in agreement with EU legislation on the general data protection regulation and decisions by the Swedish Ethical Review Authority and Region Skåne, which should be regulated in a material transfer agreement.

## Abbreviations

AD: Alzheimer’s disease
ADD: Alzheimer disease dementia
ANOVA: 2-Way repeated measures of variance
AUC: Area under the curve
Aβ: Amyloid-β
CSF: Cerebrospinal fluid
CU: Cognitively unimpaired
EDTA: Ethylenediaminetetraacetic acid
LiHep: Lithium heparin
MCI: Mild cognitive impairment
MMSE: Mini-mental state examination
MSD: Mesoscale discovery
NPH: Normal pressure hydrocephalus
PET: Positron emission tomography
p-tau: Phosphorylated tau
ROC: Receiver operating characteristic curve
RT: Room temperature
Simoa: Single-molecule array technology
VaD: Vascular dementia
