# Supplementary material for: Effects of certain pre-analytical factors on the performance of plasma phospho-tau217
Source: Alzheimers Res Ther. 2024 Feb 8;16:31. doi: 10.1186/s13195-024-01391-1 (PMC10851521; doi:10.1186/s13195-024-01391-1)
Supplement: Supplementary file 1 — Additional file 1: Fig S1. Intra-assay precision of plasma p-tau217 Lilly MSD assay. Fig S2. Plasma p-tau217 in different pre-analytical sample handling conditions. Fig S3. The accuracy of plasma p-tau217 to idetntify individuals with abnormal CSF Aβ42/Aβ40 or p-tau217 status. Table S1. Applying Youden-based cutoff of a reference condition (condition 4)a to determine accuracies, sensitivities and specificities of other conditions when identifying abnormal CSF Aβ42/Aβ40 status. Table S2. Applying Youden-based cutoff of a reference condition (condition 4)a to determine accuracies, sensitivities and specificities of other conditions when identifying abnormal CSF p-tau217 status. Table S3. Mean Differences in plasma p-tau217 levels between reference condition (condition 4)a and other conditions, first set of experiments. Table S4. Mean Differences in plasma p-tau217 levels between reference condition (Condition 2)a and other conditions, second set of experiments. Table S5. Spearman correlations between plasma p-tau217 and CSF Aβ42/Aβ40. Table S6. ROC analysis of plasma p-tau 217 for identifying abnormal CSF Aβ42/Aβ40 status. Table S7. Spearman correlations between plasma p-tau217 and CSF p-tau217. Table S8. ROC analysis of plasma p-tau 217 for identifying abnormal CSF p-tau217 status. [file 13195_2024_1391_MOESM1_ESM.docx]

**Additional File 1**

**Effects of certain pre-analytical factors on the performance of plasma phospho-tau217**

Divya Bali, Oskar Hansson, Shorena Janelidze

**Figures**

**Figure S1**

**Fig S1.** **Intra-assay precision of plasma p-tau217 Lilly MSD assay**. Coefficients of variation (CV) were plotted against mean concentrations for study samples analyzed in duplicates. Abbreviations: Aβ^+^, Amyloid-β positive; Aβ^–^, Amyloid-β negative; C, centrifugation; Cond, Condition; CSF, cerebrospinal fluid; fxt, freeze-thaw cycle; NC, non-centrifugation; RT, room temperature.

**Figure S2**

**Fig S2. Plasma p-tau217 in different pre-analytical sample handling conditions.** P-tau217 concentrations in EDTA plasma samples that were thawed at RT and underwent 1 freeze-thaw cycle without centrifugation (Cond 1) and centrifuged before the analysis (Cond 2), underwent 2 freeze thaw cycles without centrifugation (Cond 3) and centrifuged prior to analysis (Cond 4), underwent 3 freeze thaw cycles without centrifugation (Cond 5) and centrifuged before the analysis (Cond 6). Plasma samples were collected from 50 Aβ^–^ and 50 Aβ^+^ participants. P-values are from the one-way ANOVA repeated measures with FDR correction for multiple comparisons; boxes show interquartile range, the horizontal lines are the medians and the whiskers are plotted using Tukey method.

Abbreviations: Aβ^+^, Amyloid-β positive; Aβ^–^, Amyloid-β negative; EDTA, Ethylenediaminetetraacetic acid; C, centrifugation NC, non-centrifugation; RT, room temperature.

**Figure S3**

**Fig S3. The accuracy of plasma p-tau217 to idetntify individuals with abnormal CSF Aβ42/Aβ40 or p-tau217 status.** ROC curve analyses for identifying abnormal **a) CSF Aβ42/Aβ40 status b)** **CSF p-tau 217** status; (see Table S2 and S4 for sensitivity and specificity measures). Abbreviations: AUC, area under the curve; C, centrifugation; fxt, freeze-thaw cycle; NC, non-centrifugation; ROC, receiver operating characteristic; RT, room temperature.

**Table S1. Applying Youden-based cutoff of a reference condition (condition 4)^a^ to determine accuracies, sensitivities and specificities of other conditions when identifying abnormal CSF Aβ42/Aβ40 status**

| **Plasma p-tau217** | **Sensitivity %** | **Specificity %** | **Accuracy %** |
| --- | --- | --- | --- |
| Cond 1: thaw at RT, NC | 90 | 64 | 77 |
| Cond 2: thaw at RT, C | 70 | 90 | 80 |
| Cond 3: thaw on ice, NC | 86 | 82 | 84 |
| **Cond 4: thaw on ice, C** | **76** | **92** | **84** |

**^a^** Condition 4, thawing on ice and centrifugation, was used in the first paper describing the Lilly p-tau assay(1) and therefore was considered as a reference condition (highlighted in bold). Abbreviations: C, centrifugation; Cond, Condition; NC, non-centrifugation; RT, room temperature.

**Table S2. Applying Youden-based cutoff of a reference condition (condition 4)^a^ to determine accuracies, sensitivities and specificities of other conditions when identifying abnormal CSF p-tau217 status**

| Plasma p-tau217 | **Sensitivity %** | **Specificity %** | **Accuracy %** |
| --- | --- | --- | --- |
| Cond 1: thaw at RT, NC | 95.1 | 54.2 | 71 |
| Cond 2: thaw at RT, C | 87.8 | 83.1 | 85 |
| Cond 3: thaw on ice, NC | 90.2 | 72.9 | 80 |
| **Cond 4: thaw on ice, C** | **87.8** | **81.4** | **84** |

**^a^** Condition 4, thawing on ice and centrifugation, was used in the first paper describing the Lilly p-tau assay(1) and therefore was considered as a reference condition (highlighted in bold). Abbreviations: C, centrifugation; Cond, Condition; NC, non-centrifugation; RT, room temperature.

**Table S3. Mean Differences in plasma p-tau217 levels between reference condition (condition 4)^a^ and other conditions, first set of experiments**

| **Sample handling conditions** | **Aβ-** | **Aβ+** |
| --- | --- | --- |
| Cond 1: Thaw at RT, NC | 0.12 | 0.07 |
| Cond 2: Thaw at RT, C | -0.01 | -0.01 |
| Cond 3: Thaw on ice, NC | 0.06 | 0.03 |

**^a^** Condition 4, thawing on ice and centrifugation, was used in the first paper describing the Lilly p-tau assay(1) and therefore was considered as a reference condition. Abbreviations: Aβ^+^, Amyloid-β positive; Aβ ^–^, Amyloid-β negative; C, centrifugation; Cond, Condition; NC, non-centrifugation; RT, room temperature.

**Table S4. Mean Differences in plasma p-tau217 levels between reference condition (Condition 2)^a^ and other conditions, second set of experiments**

| **Sample handling conditions** | **Whole cohort** |
| --- | --- |
| Cond 1: Thaw at RT, NC, fxt-1 | -0.15 |
| Cond 3: Thaw at RT, NC, fxt-2 | -0.15 |
| Cond 4: Thaw at RT, C, fxt-2 | -0.01 |
| Cond 5: Thaw at RT, NC, fxt-3 | -0.17 |
| Cond 6: Thaw at RT, C, fxt-3 | -0.02 |

**^a^** Condition 2, thawing at RT, centrifugation and fxt-1, was considered as a reference condition because this sample handling procedure was determined to be optimal in the first set of experiments. Abbreviations: C, centrifugation; Cond, Condition; fxt, freeze-thaw cycle; NC, non-centrifugation; RT, room temperature.

**Table S5. Spearman correlations between plasma p-tau217 and CSF Aβ42/Aβ40**

| **Plasma p-tau217** | **CSF Aβ42/Aβ40**  **R (pvalue, adjusted/unadjusted)** |
| --- | --- |
| **All (n = 99)**  Cond 1: thaw at RT, NC, fxt-1  Cond 2: thaw at RT, C, fxt-1  Cond 3: thaw at RT, NC, fxt-2  Cond 4: thaw at RT, C, fxt-2  Cond 5: thaw at RT, NC, fxt-3  Cond 6: thaw at RT, C, fxt-3 | **-0.539 (<0.001 / <0.001)**  **-0.608 (<0.001 / <0.001)**  **-0.523 (<0.001 / <0.001)**  **-0.588 (<0.001 / <0.001)**  **-0.483 (<0.001 / <0.001)**  **-0.615 (<0.001 / <0.001)** |
| **Aβ^+^ (n = 50)**  Cond 1: thaw at RT, NC, fxt-1  Cond 2: thaw at RT, C, fxt-1  Cond 3: thaw at RT, NC, fxt-2  Cond 4: thaw at RT, C, fxt-2  Cond 5: thaw at RT, NC, fxt-3  Cond 6: thaw at RT, C, fxt-3 | -0.173 (0.331 / 0.231)  **-0.369 (0.019 / 0.008)**  -0.240 (0.167 / 0.093)  **-0.395 (0.012 / 0.004)**  -0.147 (0.398 / 0.309)  **-0.361 (0.020 / 0.100)** |
| **Aβ­^–^ (n = 49)***  Cond 1: thaw at RT, NC, fxt-1  Cond 2: thaw at RT, C, fxt-1  Cond 3: thaw at RT, NC, fxt-2  Cond 4: thaw at RT, C, fxt-2  Cond 5: thaw at RT, NC, fxt-3  Cond 6: thaw at RT, C, fxt-3 | 0.021 (0.889 / 0.889)  0.032 (0.873 / 0.825)  0.104 (0.571 / 0.476)  0.070 (0.712 / 0.633)  0.214 (0.230 / 0.141)  0.171 (0.331 / 0.239) |

Data are shown as Spearman correlation coefficients (p-values, adjusted/unadjusted) with significant results highlighted in bold. * One

case was missing. Abbreviations: Aβ^+^, Amyloid-β positive; Aβ^–^, Amyloid-β negative; C, centrifugation; Cond, Condition; CSF, cerebrospinal fluid; fxt, freeze-thaw cycle; NC, non-centrifugation; RT, room temperature.

**Table S6. ROC analysis of plasma p-tau 217 for identifying abnormal CSF Aβ42/Aβ40 status**

| **Plasma p-tau217** | **AUC (95% CI)** | **Sensitivity %** | **Specificity %** | **Cut-off** | **Youden´s Index** |
| --- | --- | --- | --- | --- | --- |
| Cond 1: thaw at RT, NC, fxt-1 | 0.851 (0.775, 0.927) | 86 | 77.6 | 0.281 | .636 |
| Cond 2: thaw at RT, C, fxt-1 | 0.856 (0.780, 0.933) | 80 | 83.7 | 0.201 | .637 |
| Cond 3: thaw at RT, NC, fxt-2 | 0.840 (0.761, 0.919) | 76 | 83.7 | 0.313 | .597 |
| Cond 4: thaw at RT, C, fxt-2 | 0.836 (0.755, 0.918) | 74 | 85.7 | 0.215 | .597 |
| Cond 5: thaw at RT, NC, fxt-3 | 0.838 (0.758, 0.918) | 76 | 81.6 | 0.321 | .576 |
| Cond 6: thaw at RT, C, fxt-3 | 0.876 (0.804, 0.947) | 72 | 93.9 | 0.235 | .659 |

Data are shown as AUC (95% CI). Abbreviations: Aβ^+^, Amyloid-β positive; Aβ^–^, Amyloid-β negative; AUC, area under the curve; C, centrifugation; CI, confidence interval; Cond, Condition; fxt, freeze-thaw cycle; NC, non-centrifugation; ROC, receiver operating characteristic; RT, room temperature.

**Table S7. Spearman correlations between plasma p-tau217 and CSF p-tau217**

| **Plasma p-tau217** | **CSF p-Tau217**  **R (pvalue, adjusted/unadjusted)** |
| --- | --- |
| **All (n = 99)**  Cond 1: thaw at RT, NC, fxt-1  Cond 2: thaw at RT, C, fxt-1  Cond 3: thaw at RT, NC, fxt-2  Cond 4: thaw at RT, C, fxt-2  Cond 5: thaw at RT, NC, fxt-3  Cond 6: thaw at RT, C, fxt-3 | **0.555 (<0.001 / <0.001)**  **0.677 (<0.001 / <0.001)**  **0.560 (<0.001 / <0.001)**  **0.655 (<0.001 / <0.001)**  **0.546 (<0.001 / <0.001)**  **0.721 (<0.001 / <0.001)** |
| **Aβ^+^ (n = 50)**  Cond 1: thaw at RT, NC, fxt-1  Cond 2: thaw at RT, C, fxt-1  Cond 3: thaw at RT, NC, fxt-2  Cond 4: thaw at RT, C, fxt-2  Cond 5: thaw at RT, NC, fxt-3  Cond 6: thaw at RT, C, fxt-3 | **0.382 (0.009 / 0.006)**  **0.514 (<0.001 / <0.001)**  **0.401 (0.006 / 0.004)**  **0.534 (<0.001 / <0.001)**  **0.407 (0.006 / 0.003)**  **0.581 (<0.001 / <0.001)** |
| **Aβ^–^ (n = 49) ***  Cond 1: thaw at RT, NC, fxt-1  Cond 2: thaw at RT, C, fxt-1  Cond 3: thaw at RT, NC, fxt-2  Cond 4: thaw at RT, C, fxt-2  Cond 5: thaw at RT, NC, fxt-3  Cond 6: thaw at RT, C, fxt-3 | 0.029 (0.891 / 0.842)  **0.316 (0.037 / 0.027)**  0.097 (0.571 / 0.507)  **0.305 (0.040 / 0.033)**  -0.007 (0.964 / 0.964)  **0.305 (0.040 / 0.033)** |

Data are shown as Spearman correlation coefficients (p-value, (adjusted/unadjusted) with significant results highlighted in bold. * One case was missing. Abbreviations: Aβ^+^, Amyloid-β positive; Aβ^–^, Amyloid-β negative; C, centrifugation; Cond, Condition; fxt, freeze-thaw cycle; NC, non-centrifugation; RT, room temperature.

**Table S8. ROC analysis of plasma p-tau 217 for identifying abnormal CSF p-tau217 status**

| **Plasma p-tau217** | **AUC (95% CI)** | **Sensitivity %** | **Specificity %** | **Cut-off** | **Youden´s Index** |
| --- | --- | --- | --- | --- | --- |
| Cond 1: thaw at RT, NC, fxt-1 | 0.858 (0.785, 0.931) | 90.2 | 72.4 | 0.285 | .627 |
| Cond 2: thaw at RT, C, fxt-1 | 0.910 (0.855, 0.966) | 75.6 | 93.1 | 0.241 | .687 |
| Cond 3: thaw at RT, NC, fxt-2 | 0.851 (0.775, 0.927) | 82.9 | 79.3 | 0.313 | .622 |
| Cond 4: thaw at RT, C, fxt-2 | 0.909 (0.853, 0.964) | 95.1 | 72.4 | 0.183 | .675 |
| Cond 5: thaw at RT, NC, fxt-3 | 0.863 (0.791, 0.935) | 85.4 | 75.9 | 0.315 | .612 |
| Cond 6: thaw at RT, C, fxt-3 | 0.925 (0.876, 0.974) | 80.5 | 93.1 | 0.244 | .736 |

Data are shown as AUC (95% CI). Abbreviations: AUC, area under the curve; C, centrifugation; CI, confidence interval; Cond, Condition; fxt, freeze-thaw cycle; NC, non-centrifugation; ROC, receiver operating characteristic; RT, room temperature.

**References:**

1. Mielke MM, Hagen CE, Xu J, Chai X, Vemuri P, Lowe VJ, et al. Plasma phospho-tau181 increases with Alzheimer's disease clinical severity and is associated with tau- and amyloid-positron emission tomography. Alzheimers Dement. 2018;14(8):989-97.
